# Optimal vaccination strategies using a distributed model applied to COVID-19

**DOI:** 10.1007/s10100-022-00819-z

**Published:** 2022-09-09

**Authors:** Georgi Angelov, Raimund Kovacevic, Nikolaos I. Stilianakis, Vladimir M. Veliov

**Affiliations:** 1 Institute of Statistics and Mathematical Methods in Economics, Vienna University of Technology, Vienna, Austria; 2 Department for Economy and Health, University for Continuing Education Krems, Krems a.d. Donau, Austria; 3 European Commission, Joint Research Centre (JRC), Ispra, Italy; 4 Department of Biometry and Epidemiology, University of Erlangen-Nuremberg, Erlangen, Germany

**Keywords:** Epidemics, Epidemiological model, Optimal control, SARS-CoV-2, Vaccination

## Abstract

Optimal distribution of vaccines to achieve high population immunity levels is a desirable aim in infectious disease epidemiology. A distributed optimal control epidemiological model that accounts for vaccination was developed and applied to the case of the COVID-19 pandemic. The model proposed here is nonstandard and takes into account the heterogeneity of the infected sub-population with respect to the time since infection, which is essential in the case of COVID-19. Based on the epidemiological characteristics of COVID-19 we analyze several vaccination scenarios and an optimal vaccination policy. In particular we consider random vaccination over the whole population and the prioritization of age groups such as the elderly and compare the effects with the optimal solution. Numerical results of the model show that random vaccination is efficient in reducing the overall number of infected individuals. Prioritization of the elderly leads to lower mortality though. The optimal strategy in terms of total deaths is early prioritization of those groups having the highest contact rates.

## Introduction

The control of the globally fast spreading severe acute respiratory syndrome coronavirus 2 (SARS-CoV-2) - the virus that causes coronavirus disease 2019 (COVID-19) - was more difficult than expected. Symptom based screening alone could not bring the pandemic under control. Accumulating evidence showed that SARS-CoV-2 is transmitted from persons without symptoms (Johansson et al. 2021) making infection control very difficult. In the absence of vaccines and effective drugs non-pharmaceutical interventions were the first line of defense against the pandemic. Population and individual based interventions such as isolation of infected individuals and quarantine of suspected infections were implemented widely. These measures led to a slowing down of the pandemic in the short-term. However, lifting of the measures led to resurgence of infections among people.

The remarkable speed of vaccine development became a powerful pharmaceutical tool for the control of the COVID-19 pandemic. Fast and optimal deployment of vaccination is expected to lead to a large fraction of the population with immunity, which will add to post-infection natural immunity of another part of the population. These two parallel processes will allow for reaching levels of immunity close to herd immunity, which will reduce future fatalities and severe cases. Vaccination aims at protecting individuals against infection and disease severity and reducing transmission of infected vaccinated individuals to others. The epidemiology of COVID-19 showed that severity of disease is variable among individuals, but increases with age (Davis et al. 2020). The age dependence was also observed for lethality with a strong concentration in the elderly population (Onder et al. 2020). On the other hand, the number of contacts also changes with age (Bhattacharaya et al. 2016), which influences the transmission of the pathogen. These characteristics point to the necessity of vaccine prioritization. This can be vaccination of those at highest risk of severe disease and potentially death. It can also be vaccination of those who transmit the virus most. Therefore, a fundamental question raised was how to optimize the vaccine allocation to maximize public health benefit. For instance, is it better to vaccinate younger adults or older people first? (Bubar et al. 2021) showed in a modeling approach that vaccination of younger adults may prevent the highest incidence whereas vaccination of elderly may reduce mortality. A mathematical modeling study explored different strategies for the prioritization of SARS-CoV-2 vaccines (Jentsch et al. 2021). Strategies that interrupt transmission such as vaccination of the younger population groups or vaccinating uniformly by age are more effective over time as herd immunity builds. They point to the role of timing of vaccination start. Vaccination of the elderly first was beneficial in the early stage of the pandemic. Late vaccination start would favor the employment of transmission interruption strategies over prioritizing the oldest in terms of preventing deaths. Vaccine uptake and effectiveness play also a critical role in a successful pandemic control including risks associated with early relaxation of non-pharmaceutical interventions (Moore et al. 2021). Looking at vaccine effectiveness scenario modeling showed that a vaccine with more than 50% effectiveness will lead to pandemic control, provided that a high percentage of the population is optimally vaccinated (Matrajt et al. 2021). According to this model the optimal solution to minimize deaths is to vaccinate the older population first, if vaccination effectiveness is low. For high vaccine effectiveness, transmission interruption by vaccinating high transmission age groups (usually younger people) was the optimal strategy.

In this work we adapt a previously developed epidemiological model (Kovacevic et al. 2022) to account for vaccination in order to implement several vaccination strategies and identify optimal solutions. The original distributed optimal control epidemiological model is based on a single integral equation having the number of new infections over time as the only dynamic variable. The basic approach aims at modeling the course of infection. Therefore, different subsequent sub-population compartments are considered, such as asymptomatic infected individuals, infected individuals with clinical symptoms (both isolated or not), recovered and dead individuals. The time since infection of individuals in any compartment is explicitly involved in the model, which therefore, becomes distributed and has the form of a system of integral equations. In Sect. 2 we present the fundamental characteristics of the original model and extend it to incorporate vaccination and describe vaccination scenarios. First, we consider random vaccination where susceptible individuals are randomly vaccinated with a certain rate in a certain period of time. We derive the total size of the vaccinated population and intensity before and at time *t*. We also extend the model with vaccination to account for heterogeneity of vaccination prioritized groups, we discretize the population of susceptible and recovered individuals and formulate a vaccination control policy. Minimizing the number of deaths is formulated as the control objective and the analysis of the optimization problem and is presented in Sect. 3. In Sect. 4 numerical results from plausible scenarios for COVID-19 control through vaccination are presented. These include, prioritization according to the severity of disease and risk of death that is prioritization of the elderly, transmission interruption by prioritizing younger adults, and comparison with the corresponding optimal solutions. The numerical results and the flexibility of the modified model in addressing multiple scenarios are discussed in Sect. 5.

## Description of the model

In this section we introduce the model including vaccination policies as decision variables. In that we build on the setup in Kovacevic et al. (2022), which is modified to deal with several sub-populations consisting of people having similar levels of risk, say, due to age differences, and for which vaccination could be prioritized. Therefore, we start with a short review of the original model and then introduce the necessary modifications in detail.

### The basic setup without vaccination

Kovacevic et al. (2022) split the population into compartments representing the stages of the disease that an individual may undergo during an epidemic:

- *S* – susceptible
- $I_1$ – asymptomatic, non-isolated
- $$I_2$$ – asymptomatic, isolated
- $I_3$ – symptomatic, non-isolated
- $$I_4$$ – symptomatic, isolated
- *R* – recovered
- *D* – dead

While transitions between the compartments are usually modeled by ordinary differential equations and transition rates, we use integral equations which allow to model explicitly the course of infection. Therefore, the mathematical formulation is based on the probabilities that an infected individual belongs to one or another compartment at a given infection age (*time since infection*).

These probabilities have been analyzed in the medical literature for many infectious diseases, whereas health authorities gain experience regarding the timing of quarantine and isolation measures.

The relative sizes of the compartments of infected individuals strongly depend on the infection age, as well as some key parameters such as the infectiousness and the contact rates. For this reason, the infection age plays an essential role in the model. We denote it by $\theta$. Thus an individual infected at time $t_0$ will have infection age $\theta = t - t_0$ at time $t \ge t_0$.

Kovacevic et al. (2022) assume that the environment is stationary (i.e. meteorological or behavioral changes are ignored^1^). Moreover, it is assumed there that recovered individuals remain immune after recovery. Both assumptions are kept throughout this work.

As in Kovacevic et al. (2022), we consider a finite time horizon [0, *T*], while the infection may have started earlier than time 0. It is assumed that all infected individuals either recover or die till infection age $\Theta$ (say, 40 days). The following data related to the progress of the disease along the infection age $\theta$ are required. Each of them represents the fraction of all individuals infected at the same time, having a given status ($I_1, \ldots , I_4, R, D$) at infection age $\theta \in [0,\Theta ]$:

- $$\alpha _{1}(\theta )$$ – fraction of asymptomatic non-isolated individuals ($$I_1)$$
- $\alpha _{2}(\theta )$ – fraction of asymptomatic isolated individuals ($I_2$)
- $\alpha _{3}(\theta )$ – fraction of symptomatic non-isolated individuals ($I_3$)
- $\alpha _{4}(\theta )$ – fraction of symptomatic isolated individuals ($I_4$)
- $\rho (\theta )$ – fraction of recovered individuals (*R*)
- $\mu (\theta )$ – fraction of dead individuals (*D*)

In accordance with the meaning of $\Theta$, we extend $\alpha _{k}(\theta ) = 0$, $k = 1, \ldots , 4$, and $\rho (\theta ) = \rho (\Theta )$, $\mu (\theta ) = \mu (\Theta )$ for $\theta > \Theta$.

Clearly, it must hold that for all $\theta \ge 0$.

$$\begin{aligned} \sum _{k=1}^4 \alpha _{k}(\theta ) + \rho (\theta ) + \mu (\theta ) = 1. \end{aligned}$$

The above data allow to express the sizes of each of the compartments *S*, $I_1, \ldots D$ at any time $t \ge 0$ by means of a single function of time, $y(\cdot )$, which gives the number of new infections at any given time $t \in [-\Theta , T]$. Somewhat overloading the notation, $I_k(t)$ denotes the size of the compartment $I_k$ at time $t \in [0,T]$ ($k = 1, \ldots , 4$) and similarly for *S*(*t*), *R*(*t*) and *D*(*t*). Moreover, $y^0(\theta ) := y(\theta )$ for $\theta \in [-\Theta ,0]$ are considered as known initial data, therefore they appear separately in the right-most expressions below. We have (using the extensions of the fractions introduced above for $\theta > \Theta$) that

2.1 $$\begin{aligned} I_k(t)= & {} \int _0^\Theta \alpha _k(\theta ) y(t - \theta ) \,\mathrm{d}\theta \nonumber \\= & {} \int _{-\Theta }^0 \alpha _k(t-\tau ) y^0(\tau ) \,\mathrm{d}\tau + \int _0^t \alpha _k(\theta ) y(t - \theta ) \,\mathrm{d}\theta , \quad k = 1, \ldots , 4, \end{aligned}$$

2.2 $$\begin{aligned} S(t)= & {} S(-\Theta ) - \int _{0}^{\Theta + t} y(t-\theta ) \,\mathrm{d}\theta \nonumber \\= & {} S(-\Theta ) - \int _{-\Theta }^{0} y^0(\tau ) \,\mathrm{d}\tau - \int _0^t y(t-\theta ) \,\mathrm{d}\theta , \end{aligned}$$

2.3 $$\begin{aligned} R(t)= & {} R^0 + \int _{0}^{\Theta + t} \rho (\theta ) y(t-\theta ) \,\mathrm{d}\theta \nonumber \\= & {} R^0 + \int _{-\Theta }^{0} \rho (t - \tau ) y^0(\tau ) \,\mathrm{d}\tau + \int _{0}^{t} \rho (\theta ) y(t-\theta ) \,\mathrm{d}\theta , \end{aligned}$$

2.4 $$\begin{aligned} D(t)= & {} D^0 + \int _{0}^{\Theta + t} \mu (\theta ) y(t-\theta ) \,\mathrm{d}\theta \nonumber \\= & {} D^0 + \int _{-\Theta }^{0} \mu (t - \tau ) y^0(\tau ) \,\mathrm{d}\tau + \int _{0}^{t} \mu (\theta ) y(t-\theta ) \,\mathrm{d}\theta , \end{aligned}$$

where $S(-\Theta )$ is the size of the susceptible population at time $-\Theta$, $R^0$ is the size of the compartment of individuals who have been infected before time $t = -\Theta$ and have recovered till time $t = 0$, $D^0$ is similar but for the dead individuals.

Thus, the main unknown variable in the model is *y*(*t*) – the quantity of individuals who get infected (for the first time) at time *t*. According to the definition of $\Theta$, all these individuals either recover or die at most $\Theta$ days after infection.

More data is needed for modeling the dynamics of *y*(*t*), some of which are time-dependent in order to incorporate possible distancing policy as in Kovacevic et al. (2022), namely

- *c*(*t*)    – contact rate of susceptible and recovered individuals,
- $c_k(t)$    – contact rate of individuals from compartment $I_k$, $k = 1, \ldots , 4$,
- $i_{k}(\theta )$    – infectiousness of individuals from compartment $I_k$, $k = 1, \ldots , 4$, of infection age $\theta$.

In the derivation of the equation for *y* below, natural deaths and births are ignored, which is plausible if the duration of the epidemic is not too long or the natural demographic change is slow.

It is assumed that the amount of newly infected individuals at time *t* is proportional to the number of contacting susceptibles, *S*(*t*), regarding their contact rate, *c*(*t*), and to the infectiousness per contact of the environment in which the contacts take place:

2.5 $$\begin{aligned} y(t) = \mathcal{I}(t) c(t) S(t). \end{aligned}$$

The infectiousness of the contact environment is determined on the assumption of weighted random mixing. For that we introduce the cumulative infectiousness of each of the four compartments of infected individuals:

2.6 $$\begin{aligned} J_k(t)= & {} \int _0^\Theta i_k(\theta ) \alpha _k(\theta ) y(t - \theta ) \,\mathrm{d}\theta \nonumber \\= & {} \int _{-\Theta }^0 i_k(t-\tau ) \alpha _k(t-\tau ) y^0(\tau ) \,\mathrm{d}\tau \nonumber \\&+ \int _0^t i_k(\theta ) \alpha _k(\theta ) y(t - \theta ) \,\mathrm{d}\theta , \quad k = 1, \ldots , 4. \end{aligned}$$

Then $\mathcal{I}(t)$ takes the form

2.7 $$\begin{aligned} \mathcal{I}(t) = \frac{P(t)}{Q(t)}, \end{aligned}$$

where

$$\begin{aligned} P(t) = \sum _{k=1}^4 c_k(t) J_k(t), \qquad Q(t) = c(t) S(t) + c(t) R(t) + \sum _{k=1}^4 c_k(t) I_k(t) \end{aligned}$$

are the cumulative infectiousness of the contact environment and the total number of contacts per unit of time, respectively. Substituting the obtained expressions for $\mathcal{I}$, *P* and *Q* in (2.5) and using (2.1), (2.2) and (2.6), we obtain the equation

2.8 $$\begin{aligned} y(t) = c(t) \frac{ \left( d_1(t) + \int _{0}^{t} p(t,\theta ) y(t-\theta ) \,\mathrm{d}\theta \right) \,\left( d_2 - \int _{0}^{t} y(t-\theta ) \,\mathrm{d}\theta \right) }{d_3(t) +\int _{0}^{t} q(t,\theta ) y(t-\theta ) \,\mathrm{d}\theta }, \end{aligned}$$

where

$$\begin{aligned} p(t,\theta ):= & {} \sum _{k=1}^4 c_k(t) i_k(\theta ) \alpha _k(\theta ), \qquad q(t,\theta ) := \sum _{k=1}^4 c_k(t) \alpha _k(\theta ) + c(t) (\rho (\theta ) - 1), \\ d_1(t):= & {} \int _{-\Theta }^0 p(t,t-\tau ) y^0(\tau ) \,\mathrm{d}\tau , \qquad d_2 := S(-\Theta ) - \int _{-\Theta }^0 y^0(\tau ) \,\mathrm{d}\tau , \\ d_3(t):= & {} c(t) (S(-\Theta ) + R^0) + \int _{-\Theta }^0 q(t,t-\tau ) y^0(\tau ) \,\mathrm{d}\tau . \end{aligned}$$

Notice that $y(\cdot )$ is the only unknown variable in the model and (2.8) is an evolutionary integral equation for its dynamic. All other functions that directly or indirectly appear in (2.8) are data that are assumed to be known.

We mention that the model can be further extended, say by including hospitalized individuals, hospitalized in intensive care, etc., provided that data similar to $\alpha _k$ are available for these compartments. Such extensions, however, do not change the structural form of the transmission dynamics (2.8).

### Modeling random vaccination

Using the basic notations from the previous subsection we extend the model step by step. First, we consider the simplest vaccination scenario where susceptible individuals are randomly vaccinated with a certain intensity and in a certain period of time. The vaccinated individuals are treated in the same way as recovered ones. It is assumed that the immunity after vaccination, as well as after recovery, is permanent (considering waning immunity will be subject of future work). Also, if there is a delay between vaccination and immunity or several doses have to be given over time, we model the effective vaccination as occurring at the time when immunity is reached.

Vaccination begins at time $t_v \ge 0$. Since we assume permanent immunity, the vaccination becomes redundant after a while. We assume that it ends at time ${\bar{t}}_v > t_v$, and that in the period of time $[t_v, {\bar{t}}_v] \subset [0,T]$ there are enough vaccines, vaccination facilities, and individuals willing to be vaccinated, so that one can achieve a given vaccination intensity *v*(*t*) at any time $t \in [t_v, {\bar{t}}_v]$. This means that the total size of the vaccinated population prior to time $t \ge 0$ is

2.9 $$\begin{aligned} w(t) := \left\{ \begin{array}{ll} 0 &{} \text{ for } t \in [0, t_v], \\ \int _{t_v}^{t} v(s) \,\mathrm{d}s&{} \text{ for } t \in [t_v, {\bar{t}}_v], \\ \int _{t_v}^{{\bar{t}}_v} v(s) \,\mathrm{d}s &{} \text{ for } t > {\bar{t}}_v. \end{array} \right. \end{aligned}$$

Then regarding the vaccination, the basic model remains essentially the same, with the following modifications: the expressions for *S*(*t*) and *R*(*t*) in (2.2) and (2.3) are replaced with

2.10 $$\begin{aligned} S(t)= & {} S(-\Theta ) - \int _{-\Theta }^{0} y^0(\tau ) \,\mathrm{d}\tau - \int _0^t y(t-\theta ) \,\mathrm{d}\theta - w(t), \end{aligned}$$

2.11 $$\begin{aligned} R(t)= & {} R^0 + \int _{-\Theta }^{0} \rho (t - \tau ) y^0(\tau ) \,\mathrm{d}\tau + \int _{0}^{t} \rho (\theta ) y(t-\theta ) \,\mathrm{d}\theta + w(t), \end{aligned}$$

and this expression for *S*(*t*) has to be used in (2.5). The multiplier $\mathcal{I}(t)$ does not change, because *P*(*t*) depends on $J_k(t)$ only, and the terms *w*(*t*) cancel in the expression for *Q*(*t*). Since the vaccinated individuals are treated as recovered, the basic Eq. (2.8) remains the same, with the only difference that $d_2$ has to be replaced with ${\bar{d}}_2(t)$, where

$$\begin{aligned} {\bar{d}}_2(t) := S(-\Theta ) - \int _{-\Theta }^0 y^0(\tau ) \,\mathrm{d}\tau - w(t). \end{aligned}$$

In this modification of the model it is implicitly assumed that the vaccinated individuals are chosen “randomly”, that is, disregarding the possible heterogeneity of the population with respect to disease-related mortality and contact rates. An enhancement of the model, taking into account the heterogeneity of the population follows in the next subsection.

### Extension of the vaccination model involving population heterogeneity

In order to enable prioritized vaccination of certain population groups we split the population compartments *S*, *R* and $I_k$ in the basic model into *m* groups:

$$\begin{aligned} S(t) = \sum _{j=1}^m S_j(t), \quad R(t) = \sum _{j=1}^m R_j(t), \quad I_k(t) = \sum _{j=1}^m I_{k,j}(t), \quad k = 1, \ldots , 4. \end{aligned}$$

The affiliation of a susceptible individual to a group is preserved after infection, recovery or vaccination. Each of the groups has specific parameters $c^j(t)$, $c_k^j(t)$, $\rho ^j(\theta )$, $\mu ^j(\theta )$, $\alpha _k^j(\theta )$, $j=1,\ldots , m$, $k= 1, \ldots , 4$. For example, $\alpha _1^j(\theta )$ is the fraction of asymptomatic non-isolated individuals of infection age $\theta$ in the group *j*, among all the individuals in the same group and infection age $\theta$, $c_1^j(t)$ is the contact rate of non-isolated infected individuals of type *j* during the asymptomatic period, etc. Clearly, the identities

$$\begin{aligned} \sum _{k=1}^4 \alpha _{k}^j(\theta ) + \rho ^j(\theta ) + \mu ^j(\theta ) = 1, \quad j=1, \ldots , m, \end{aligned}$$

have to be fulfilled. We also split the new cases into *m* groups $y_1(t), \ldots , y_m(t)$, $t \in [-\Theta , T]$, where $y_j^0(t) = y_j(t)$, $t \in [-\Theta ,0]$ are known initial data for each group. The meaning of $S_j(-\Theta )$ and $R_j^0$ is clear from the notations. The group-specific form of expression (2.1) is straightforward:

$$\begin{aligned} I_{k,j}(t) = \int _0^\Theta \alpha _k^j(\theta ) y_j(t - \theta ) \,\mathrm{d}\theta = \int _{-\Theta }^0 \alpha _k^j(t-\tau ) y^0_j(\tau ) \,\mathrm{d}\tau + \int _0^t \alpha _k^j(\theta ) y_j(t - \theta ) \,\mathrm{d}\theta . \end{aligned}$$

Following the group splitting, we denote by $v^j(t)$ the intensity of vaccination at time *t* of members of group *j*. Correspondingly, $w_j(t)$ denotes the size of the vaccinated till time *t* subpopulation of group *j*. Then the formulae (2.6) and (2.10) take the group-wise form

2.12 $$\begin{aligned} J_k^j(t)= & {} \int _0^\Theta i_k^j(\theta ) \alpha _k^j(\theta ) y_j(t - \theta ) \,\mathrm{d}\theta \nonumber \\= & {} \int _{-\Theta }^0 i_k^j(t-\tau ) \alpha _k^j(t-\tau ) y^0_j(\tau ) \,\mathrm{d}\tau + \int _0^t i_k^j(\theta ) \alpha _k^j(\theta ) y_j(t - \theta ) \,\mathrm{d}\theta ,\qquad \quad \end{aligned}$$

2.13 $$\begin{aligned} S_j(t)= & {} S_j(-\Theta ) - \int _{-\Theta }^{0} y_j^0(\tau ) \,\mathrm{d}\tau - \int _0^t y_j(t-\theta ) \,\mathrm{d}\theta - w_j(t). \end{aligned}$$

Assuming random mixing of all groups, we have similar to (2.5) and (2.7) that

2.14 $$\begin{aligned} y_j(t) = \frac{P(t)}{Q(t)} c^j(t) S_j(t), \end{aligned}$$

where *P*(*t*) and *Q*(*t*) have the same meaning as in the basic model, but are now given by

$$\begin{aligned} P(t)= & {} \sum _{k=1}^4 \sum _{j=1}^m c_k^j(t) J_k^j(t),\\ Q(t)= & {} \sum _{j=1}^m c^j(t) (S_j(t) + R_j(t) ) + \sum _{k=1}^4 \sum _{j=1}^m c_k^j(t) I_{k,j}(t). \end{aligned}$$

Substituting the above expressions in (2.14) we obtain the system

2.15 $$\begin{aligned} y_s(t)= & {} c^s(t) \frac{ \left( d_1(t) + \int _{0}^{t} \sum _{j=1}^m p^j(t,\theta ) y_j(t-\theta ) \,\mathrm{d}\theta \right) \,\left( d_2^s - w_s(t) - \int _{0}^{t} y_s(t-\theta ) \,\mathrm{d}\theta \right) }{ d_3(t) +\int _{0}^{t} \sum _{j=1}^m q^j(t,\theta ) y_j(t-\theta ) \,\mathrm{d}\theta }, \quad \nonumber \\ s= & {} 1, \ldots ,m, \end{aligned}$$

where

2.16 $$\begin{aligned} p^j(t,\theta ):= & {} \sum _{k=1}^4 c_k^j(t) i_k^j(\theta ) \alpha _k^j(\theta ), \qquad q^j(t,\theta ) := \sum _{k=1}^4 c_k^j(t) \alpha _k^j(\theta ) + c^j(t) (\rho ^j(\theta ) - 1), \nonumber \\ d_1(t):= & {} \int _{-\Theta }^0 \sum _{j=1}^m p^j(t,t-\tau ) y_j^0(\tau ) \,\mathrm{d}\tau , \qquad d_2^s := S_s(-\Theta ) - \int _{-\Theta }^0 y_s^0(\tau ) \,\mathrm{d}\tau , \nonumber \\ d_3(t):= & {} \sum _{j=1}^m c^j(t) (S_j(-\Theta ) + R_j^0) + \int _{-\Theta }^0 \sum _{j=1}^m q^j(t,t-\tau ) y_j^0(\tau ) \,\mathrm{d}\tau . \end{aligned}$$

A vaccination policy is any allocation of the available vaccination capacity, ${\bar{v}}(t)$, to the groups of susceptibles. That is, any *m*-tuple $(v_1(\cdot ), \ldots v_m(\cdot ))$ of measurable functions $v_j$ satisfying the constraints

2.17 $$\begin{aligned} v_j(t) \ge 0, \qquad \sum _{j=1}^m v_j(t) \le {\bar{v}}(t), \end{aligned}$$

is considered as feasible control (vaccination) policy. Then

2.18 $$\begin{aligned} w_j(t) = \int _0^t v_j(\tau ) \,\mathrm{d}\tau \end{aligned}$$

is the number of vaccinated before time *t* individuals from the *j*-th group, which appears in (2.15).

As mentioned above, Eq. (2.15) is based on the assumption of random mixing over all (pooled) groups. This approach can be easily enhanced by considering mixing with various contact rates between and within groups. To this end, we consider contact rates $c^{ij}(\cdot )$ for the susceptible (recovered) individuals in group *i* with individuals of group *j*. In addition, contact rates $c^{ij}_k(\cdot )$ for the infected compartments are needed. In the simplest case, they can be calculated as $c^{ij}_k(\cdot )=\eta _k c^{ij}(\cdot )$, where $\eta _k$ is a group specific factor, modeling the reduction of contacts.

Such an approach can be used to model the relations between different behavioral groups in more detail, accounting for the fact that the depth of relations between varying pairs of groups may substantially differ. Elderly people in nursing homes or children are examples of groups with intense contacts within the group and much lower outside the group.

The equations for the newly infected within groups become

$$\begin{aligned} y_s(t)= & {} \left( d_2^s - w_s(t) - \int _{0}^{t} y_s(t-\theta ) \,\mathrm{d}\theta \right) \\&\cdot \sum _{j=1}^m c^{sj}(t) \frac{ d^{js}_1(t) + \int _{0}^{t} p^{js}(t,\theta ) y_j(t-\theta ) \,\mathrm{d}\theta }{ d^{js}_3(t) +\int _{0}^{t} q^{js}(t,\theta ) y_j(t-\theta ) \,\mathrm{d}\theta }, \quad s=1, \ldots ,m, \end{aligned}$$

where

$$\begin{aligned} p^{js}(t,\theta ):= & {} \sum _{k=1}^4 c_k^{js}(t) i_k^j(\theta ) \alpha _k^j(\theta ), \quad q^{js}(t,\theta ) := \sum _{k=1}^4 c_k^{js}(t) \alpha _k^j(\theta ) + c^{js}(t) (\rho ^j(\theta ) - 1), \\ d^{js}_1(t):= & {} \int _{-\Theta }^0 p^{js}(t,t-\tau ) y_j^0(\tau ) \,\mathrm{d}\tau , \quad d_2^s := S_s(-\Theta ) - \int _{-\Theta }^0 y_s^0(\tau ) \,\mathrm{d}\tau , \\ d_3^{js}(t):= & {} c^{js}(t) (S_j(-\Theta ) + R_j^0) + \int _{-\Theta }^0 q^{js}(t,t-\tau ) y_j^0(\tau ) \,\mathrm{d}\tau . \end{aligned}$$

The main drawback of the extension involving various contact rates between and within groups, is that it requires more data that are hardly available. Therefore we will not use it in the present paper.

## Optimal vaccination policy

As explained in the preceding section, the vaccination intensities, $v_j(t)$, will be used as control policies. Feasible controls (allocation policies for the available vaccines) are the measurable functions satisfying the constraints (2.17). The controls influence the dynamics (2.15) through the cumulative quantities $w_j$ in (2.18).

Various control objectives may be reasonable. One goal of the vaccination control may be to ensure (together with additional policies such as social distancing) that the number of individuals needing hospitalization does not exceed the capacity of the hospitals. This constraint takes the form

3.1 $$\begin{aligned} \int _{-\Theta }^0 \sum _{j=1}^m h_4^j(t-\tau ) y_j^0(\tau ) \,\mathrm{d}\tau + \int _{0}^t \sum _{j=1}^m h_4^j(\theta ) y_j(t-\theta ) \,\mathrm{d}\theta \le H(t), \end{aligned}$$

where $h_4^j(\theta )$ is the fraction of symptomatic isolated individuals of infection age $\theta$ who need hospitalization, and *H*(*t*) is the capacity of hospitals at time *t*. Clearly, the first term in the above expression is just a known number.

The constraint (3.1) may be regarded in the context of optimization involving contact restrictions, where the economic consequences of the latter should be minimized. The economic consequences of “lock-down” have been recently investigated in several papers, e.g. (Acemoglu et al. 2000; Bloom et al. 2000; Kovacevic et al. 2022; Caulkins et al. 2021). However, in the present paper we focus on the purely health benefits of vaccination, therefore we set the minimization of deaths as objective. Formally, the problem reads as

$$\begin{aligned} \min _v \; \int _0^T \left[ \int _{-\Theta }^0 \sum _{j=1}^m \mu ^j(t-\tau ) y_j^0(\tau ) \,\mathrm{d}\tau + \int _{0}^t \sum _{j=1}^m \mu ^j(\theta ) y_j(t-\theta ) \,\mathrm{d}\theta \right] \,\mathrm{d}t. \end{aligned}$$

We remind that $\mu ^j(\theta )$ is the fraction of dead individuals of infection age $\theta$ in the *j*-th group, among all infected individuals of the same infection age and group. Since the first term in the above expression is independent of the control, we can reformulate the problem as

3.2 $$\begin{aligned} \min _v \; \int _0^T \int _{0}^t \sum _{j=1}^m \mu ^j(\theta ) y_j(t-\theta ) \,\mathrm{d}\theta \,\mathrm{d}t. \end{aligned}$$

subject to the state dynamics (2.15) and the constraints (2.17)–(2.18).

For convenience we reformulate the problem in a more general way as follows. The state is a function $y:[0,T] \rightarrow {\mathbb {R}}^m$, the control is a measurable function $v: [0,T] \rightarrow {\mathbb {R}}^m$. We also define an aggregated state $z : [0,T] \rightarrow {\mathbb {R}}^{n}$ and control $w:[0,T] \rightarrow {\mathbb {R}}^m$. The controlled dynamics is described by the equations

3.3 $$\begin{aligned} y(t)= & {} f(t, z(t),w(t)), \end{aligned}$$

3.4 $$\begin{aligned} z(t)= & {} \int _0^t P(t,\theta ) y(t-\theta ) \,\mathrm{d}\theta , \end{aligned}$$

3.5 $$\begin{aligned} w(t)= & {} \int _0^t v(\tau ) \,\mathrm{d}\tau , \end{aligned}$$

where $f: [0,T] \times {\mathbb {R}}^n \times {\mathbb {R}}^m \rightarrow {\mathbb {R}}^m$, $P: [0,T] \times [0,T] \rightarrow {\mathbb {R}}^{n \times m}$. In addition, there are control constraints of the form

3.6 $$\begin{aligned} v_j(t) \ge 0, \qquad \sum _{j=1}^m v_j(t) \le {\bar{v}}, \end{aligned}$$

where ${\bar{v}} > 0)$.^2^ The objective functional to be minimized will have the form

3.7 $$\begin{aligned} F(v) := \int _0^T \int _{0}^t \langle l(t,\theta ), y(t-\theta ) \rangle \,\mathrm{d}\theta \,\mathrm{d}t, \end{aligned}$$

where $l : [0,T] \times [0,T] \rightarrow {\mathbb {R}}^{m}$ is continuous and $\langle \cdot , \cdot \rangle$ denotes the scalar product in ${\mathbb {R}}^m$. Thus we encounter an optimal control problem for a specific class of integro-differential systems.

Further we denote by $\mathcal{V}$ the set of all measurable functions (feasible controls) *v* satisfying (3.6), and by $\mathcal{W}$ the set of all functions *w* resulting from (3.5) for some $v \in \mathcal{V}$. In addition, denote by *W* the set of all values that *w*(*t*) may take when $w \in \mathcal{W}$.

Obviously, our epidemiological problem is a special case of problem (3.3)–(3.7). Indeed, set $n=m+2$. For convenience we denote the components of *z* by $(z_1, z_2^1, \ldots , z_2^m, z_3)$. Then we recast the function $f_s$ in (2.15) as

3.8 $$\begin{aligned} f_s(t,z,w) = c^s(t) \frac{(d_1(t) + z_1(t))(d_2^s(t) - z_2^s))}{d_3(t) + z_3}, \qquad s = 1, \ldots , m, \end{aligned}$$

where *z* is given by (3.4) with the following matrix-function *P* (skipping the arguments in (2.16))

$$\begin{aligned} P = \left( \begin{array}{cccc} p^1 &{}p^2&{} \ldots &{}p^m \\ 1 &{}0 &{}\ldots &{}0 \\ 0 &{}1 &{}\ldots &{}0 \\ \ldots &{}\ldots &{}\ldots &{}\ldots \\ 0 &{}0 &{}\ldots &{}1 \\ q^1 &{}q^2&{} \ldots &{}q^m \end{array} \right) . \end{aligned}$$

The objective integrand is

$$\begin{aligned} l(t,\theta ) = l(\theta ) := (\mu ^1(\theta ), \ldots , \mu ^m(\theta ))^\top , \end{aligned}$$

where ${}^\top$ means transposition.

In order to facilitate the analysis of problem (3.3)–(3.7), in the next paragraphs we introduce a few notations and make some assumptions.

The notations $L^2$ and $L^\infty$ will be used for the space of all measurable (vector-) functions on [0, *T*] that are square integrable, respectively essentially bounded. The norm in $L^\infty$ will be denoted by $\Vert \cdot \Vert _\infty$, while a weighted norm in $L^2$ will be used in the proof of the next proposition 3.1.

For any $w \in \mathcal{W}$, let $\mathcal{F}_w$ be a map on $L^2$ with values in the space of all measurable functions on [0, *T*] defined as

$$\begin{aligned} \mathcal{F}_w(y)(t) = f\left( t, \int _0^t P(t,\theta ) y(t-\theta ) \,\mathrm{d}\theta , w(t)\right) , \quad y \in L^2. \end{aligned}$$

We assume that there exists a closed bounded convex subset $\mathcal{Y}$ of $L^2$ which is invariant with respect to any $\mathcal{F}_w$, meaning that $\mathcal{F}_w(y) \in \mathcal{Y}$ for every $w \in \mathcal{W}$ and $y \in \mathcal{Y}$. Moreover, we assume that the function *f*(*t*, *z*, *w*) is continuous in *t* and differentiable in (*z*, *w*) with Lipschitz continuous derivatives (uniformly with respect to $t \in [0,T]$) in a neighborhood of the set $W \times Z$, where

$$\begin{aligned} Z := \left\{ z = \int _0^t P(t,\theta ) y((t-\theta ) \,\mathrm{d}\theta \text{ for } \text{ some } y \in \mathcal{Y} \text{ and } \text{ some } t \in [0,T] \right\} . \end{aligned}$$

Before we begin with the analysis of problem (3.3)–(3.7), we comment on the verification of the assumptions made above for the particular optimal vaccination problem (3.2), (2.15), (2.17)–(2.18). There are two problems in the verification of the assumptions concerning the function *f* in (3.8). One is to ensure that the second multiplier in the numerator remains positive (that is, no group totally dies out), the second is that the denominator does not approach zero (that is, the amount of contacting individuals stays above certain positive level). Both requirements are completely plausible from epidemiological point of view, but require formal assumptions that we skip in this paper; essentially they are similar to those formulated in Sect. 2.2 of Kovacevic et al. (2022).

Now we return to the general problem (3.3)–(3.7).

### Proposition 3.1

On the assumptions made, for every feasible control $v \in \mathcal{V}$ the system (3.3)–(3.5) has a unique solution $y \in \mathcal{Y}$.

### Proof

As usual, the proof uses the contraction mapping theorem; we present it in detail because system (3.3)–(3.4) does not fit to the standard format Volterra integral equations.

In the space $L^\infty$ we consider the weighted norm

$$\begin{aligned} \Vert y \Vert _\nu := \int _0^T e^{-\nu t} |y(t)| \,\mathrm{d}t. \end{aligned}$$

For an arbitrarily fixed $v \in \mathcal{V}$ and the corresponding *w* in (3.5) we shall prove that the map $\mathcal{F}_w: \mathcal{Y}\rightarrow \mathcal{Y}$ is contractive with respect to this norm, provided that the number $\nu$ is chosen sufficiently large. This implies the existence and uniqueness claims of the proposition.

Let *L* be the Lipschitz constant of *f* with respect to *z* in the set *Z*. For any $y_1, y_2 \in \mathcal{F}_w$ we have

$$\begin{aligned}&\Vert \mathcal{F}_w(y_1) - \mathcal{F}_w(y_2) \Vert _\nu ^2\\&\quad = \int _0^T e^{-\nu t} | \mathcal{F}_w(y_1)(t) - \mathcal{F}_w(y_2)(t)|^2 \,\mathrm{d}t \\&\quad \le L^2 \int _0^T e^{-\nu t} | z_1(t) - z_2(t)|^2 \,\mathrm{d}t \\&\quad \le L^2 \int _0^T e^{-\nu t} \left( \int _0^t | P(t,\theta ) (y_1(t-\theta ) - y_2(t-\theta ))| \,\mathrm{d}\theta \right) ^2 \,\mathrm{d}t \\&\quad \le L^2 \Vert P \Vert _\infty ^2 \int _0^T e^{-\nu t} \left( \int _0^t |y_1(\tau ) - y_2(\tau )| \,\mathrm{d}\tau \right) ^2 \,\mathrm{d}t \\&\quad \le L^2 \Vert P \Vert _\infty ^2 \int _0^T e^{-\nu t} \left( \int _0^t \left( e^{\frac{\nu \theta }{2}}\right) \left( e^{-\frac{\nu \theta }{2}} |y_1(\tau ) - y_2(\tau )| \right) \,\mathrm{d}\tau \right) ^2 \,\mathrm{d}t \\&\quad \le L^2 \Vert P \Vert _\infty ^2 \int _0^T e^{-\nu t} \int _0^t e^{\nu \tau } \,\mathrm{d}\tau \int _0^t e^{- \nu \tau } |y_1(\tau ) - y_2(\tau )|^2 \,\mathrm{d}\tau \,\mathrm{d}t \\&\quad \le L^2 \Vert P \Vert _\infty ^2 \frac{T}{\nu } \Vert y_1 - y_2 \Vert _\nu ^2. \end{aligned}$$

Clearly, by choosing $\nu$ sufficiently large we may ensure that the operator $\mathcal{F}_w$ is contractive on $\mathcal{Y}$ with respect to the norm $\Vert \cdot \Vert _\nu$ in $L^2$. Thus it has a unique fixed point $y \in \mathcal{F}$, and it (together with *z* given by (3.4)) is the unique solution of system (3.3)–(3.4) in $\mathcal{Y}$ for the fixed *v*. $\square$

### Proposition 3.2

The optimal control problem (3.3)–(3.7) has a solution.

### Proof

If $\{(y^k, z^k,w^k,v^k) \}_k$ is a minimizing sequence with $y^k \in \mathcal{Y}$ and $v^k \in \mathcal{V}$ then, due to the weak compactness of $\mathcal{Y}$ and $\mathcal{V}$, one may extract a subsequence $\{(y^k, v^k) \}_k$ which is weakly convergent in $L^2$ to some $(y,v) \in \mathcal{Y}\times \mathcal{V}$. Then due to (3.4) and (3.5), the sequences $\{z^k \}_k$ and $\{w^k\}_k$ converge point-wise to $z(t) : = \int _0^t P(t,\theta ) y(t - \theta ) \,\mathrm{d}\theta$ and $w(t) : = \int _0^t v(\tau ) \,\mathrm{d}\tau$, respectively. Then $f(t,z^k(t), w^k(t))$ converges point-wise to *f*(*t*, *z*(*t*), *w*(*t*)). This implies that $\{(y, z,w,v)$ solves (3.3)–(3.5) and must be an optimal solution, because one can pass to the limit in (3.7). $\square$

It turns out that the functional *F* in (3.7) is Fréchet differentiable in $L^2$, which is to be expected due to the linear structure of the problem with respect to *v* and *y*. Details of the derivation of the next result are presented in Kovacevic et al. (2022), Appendix.

### Proposition 3.3

On the assumptions made above, the objective functional *F*(*v*) is Fréchet differentiable in $L^2$ at every $v \in \mathcal{V}$, and its derivative has the representation

$$\begin{aligned} F'(v)(t) = f_w(t,z(t),w(t))^\top \lambda (t), \end{aligned}$$

where *w* and *z* correspond to *v*, and $\lambda$ is the unique solution of the Volterra integral equation

3.9 $$\begin{aligned} \lambda (t) = \int _t^T [P(\tau ,\tau -t)^\top f_z(t,z(t),w(t)) \lambda (\tau ) + l(\tau ,\tau -t)] \,\mathrm{d}\tau . \end{aligned}$$

Now, let $({{\hat{y}}}, {{\hat{z}}}, {{\hat{w}}}, {{\hat{v}}})$ with ${{\hat{y}}} \in \mathcal{Y}$ and ${{\hat{v}}} \in \mathcal{V}$ be an optimal solution of problem (3.3)–(3.7). Denote by *V* the set of feasible control values, that is $V = \{ v \in {\mathbb {R}}^m : \,v_j \ge 0, \, \sum _{j=1}^m v_j \le {\bar{v}}\}$. As a consequence of Proposition 3.3 we obtain the following necessary optimality condition.

### Corollary 3.4

If $({{\hat{y}}}, {{\hat{z}}}, {{\hat{w}}} , {{\hat{v}}})$ is an optimal solution of problem (3.3)–(3.7), then there exists a unique solution ${\hat{\lambda }} \in L^\infty [0,T]$ of (3.9), with (*z*, *w*) replaced with $({{\hat{z}}}, {{\hat{w}}})$, such that

3.10 $$\begin{aligned} f_w ({{\hat{z}}}(t),{{\hat{w}}}(t))^\top {\hat{\lambda }}(t) \in -N_{V}({{\hat{v}}}(t)) \quad \text{ for } \text{ a.e } t \in [0,T], \end{aligned}$$

where $N_V(v)$ is the usual normal cone to the convex set *V* at the point *v*.

We mention that the existence of the derivative of the objective functional with respect to the control and the constructive way of its calculation open the door for efficient numerical algorithms, such as the gradient projection method in the control space. However, in the present paper we do no discuss the numerical aspects of the problem.

Let us return to the epidemiological problem (3.2) subject to the state dynamics (2.15) and the constraints (2.17)–(2.18). Since $\Delta y(t) := f_w(t,z(t),w(t)) \Delta w(t)$ is the first order increment of newly infected individuals resulting from an increment $\Delta w(t)$ of the number of vaccinated individuals, the expression for $F'(v)(t)$ in Proposition 3.3 means that $\lambda (t)$ is the marginal “cost” (increment of dead people) resulting from a unit increase of the new infections at time *t*. In this sense, ${\hat{\lambda }}(t)$ is the “shadow price” of new infections at time *t* at the optimal solution.

Notice that the set of feasible control values, *V*, is a simplex, at any vertex of which at most one of the components $v_j$ may be non-zero and equals ${\bar{v}}$. Since the variational inequality (3.10) is linear (meaning that the left-hand side is independent of *v*), one can expect that the optimal control has a switching structure: at any time only members of one group are vaccinated with the maximal intensity ${\bar{v}}$. Of course, presence of singular arcs (where the optimality condition does not uniquely define a control) cannot be excluded a priori. However, in our experiments with the COVID-19 model we always encounter purely switching structure of the optimal control (see next section).

### Remark 3.5

As mentioned above, the objective functional (3.2) has a purely “humanitarian” meaning. The cost of the vaccination is ignored, as well as the economic benefits from the reduction of the diseased population due to vaccination. Including a strongly convex cost of vaccination with a “small” weighting coefficient can be viewed as Tikhonov regularization of the problem. The optimal vaccination policy in this case will no longer be of bang-bang type, however, it will approximate in the space $L^2$ the optimal policy for the case of zero vaccination cost.

## Case studies

We use the presented COVID-19 vaccination model to compare the optimal vaccination strategy for age groups with several plausible vaccination strategies. The model is parametrized based on available epidemiological and sociological data as described below, see also (Kovacevic et al. 2022) for a deeper discussion of the course of infection.

### Basic parametrization

In our vaccination model we consider five age groups of different individuals: $0-18$ years, $18-30$ years, $30-65$ years, $65-80$ years and over 80 years. The considered age structure depicts one young age group, two groups of working people and two groups of retired people. This allows us to use groups specific sizes, contact and mortality rates.

The size of these groups is derived from official information on five years groups at Statistics Austria (2020). The different contact rates of our groups were modeled based on Bhattacharaya et al. (2016) Fig. 1, depicting the average number of alters for egos of varying age. For each of the considered age groups, the proportion of cumulated alters (compared with cumulated alters over 100 years) is used as a modifying factor for the overall contact rate *c*. Here it is assumed that the contact rate is roughly proportional to the age specific number of alters.

For characterizing the mortality, we use (Signorelli and Odone 2020). Note that the age groups in the mentioned paper are different from the groups in our case study. Therefore, the mortality is recalculated by accounting for the size of five years subgroups within our subgroup population. Here, it has to be assumed that mortality is constant over all five years groups within any age group.

In our model only individuals with symptoms may die. In order to describe the morality depending on time since infection $\theta$, we use the mortality function estimated by Verity et al. (2020). This raw mortality function is normalized such that the area below the function is equal to one. The functions $\mu ^j$ then can be constructed by multiplying the normalized mortality function by the proportion of eventually symptomatic individuals within the class of infected in group *j* and with the proportion of fatally ill individuals within the class of eventually symptomatic individuals in group *j*.

In Table 1 we provide the values describing the group specific parameters. Note that the contact rates in Table 1 are used as factors: they are used for deriving the group contact rate by multiplication with the overall contact rate *c* of the population.

**Fig. 1:**
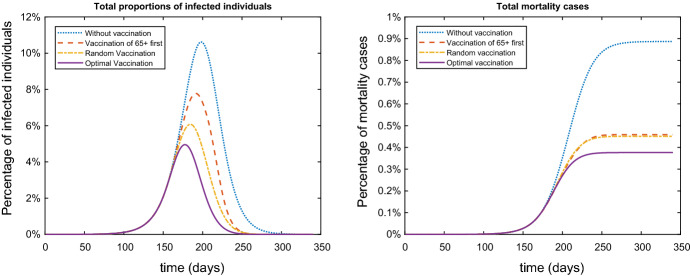
Comparison between the percentage of infected individuals (left) and comparison between mortality cases (right) with different vaccination strategies

**Table 1:** Parameters for age specific groups

Groups	0–18	18–30	30–65	65–80	80+
Fraction of population	0.1933	0.1257	0.4907	0.1370	0.0532
Contact rate (factor)	0.5271	1.6209	1.2250	0.7982	0.7011
Case fatality rate	0.0010	0.0010	0.0369	0.2232	0.3460

The class of infected individuals at given time *t* consists of individuals who show symptoms over some time and of completely asymptomatic infected individuals, who recover without having shown any symptoms.

Finally, the available epidemiological information in the course of the infection can be used to construct parameter functions $\alpha _k^j, \mu ^j, \rho ^j$ and $c^j, j=1,5, k=1,4$, similar to the parameter functions $\alpha _k, \mu , \rho$ and *c*,  described in Kovacevic et al. (2022).

In Table 2 we provide values of the parameters that are used for the numerical experiments and are different of the previously used in Kovacevic et al. (2022).

**Table 2:** Parameter values

Overall probability of infection per contact	0.1429
Overall contact rate	12.978
Reduction of contact rate for isolated individuals	0.09
Reduction of contact rate for non isolated symptomatic individuals	0.97
Infected without symptoms	0.425
Percentage of isolated asymptomatic cases	0.60
Percentage of isolated symptomatic cases	0.85

### Numerical results

Below we present numerical results showing the evolution of the epidemic without and with implementation of different vaccination policies. The vaccines at time *t* are only administered to individuals that do not show symptoms, i.e. individuals of the different age groups who do not belong to classes $I_3$ and $I_4$.

We consider the following vaccination scenarios.

Case 1: no vaccination is applied during the duration of the disease.

Case 2: random vaccination is administered to the population with an exception of the age group $0-18$.

Case 3: the vaccination begins with the vaccination of the elder population, that is, the two age groups of $65-80$ and $80+$. After all suitable for vaccination individuals of these groups are vaccinated, the vaccination continues by applying the obtained by numerical optimization vaccine policy to the rest of the groups.

Case 4: the numerical calculated optimal vaccination strategy is applied.

Additional experiments regarding the optimal vaccination order in our model (Case 4) are also presented, exploring the importance of the contact rates of the groups.

For all scenarios we consider a time horizon from 0 to 455 days. The time interval $[t_v, \bar{t}_v]$ on which the vaccination control *v*(*t*) is applied is [150, 250].One can easily extend or change the vaccination administration interval $[t_v, \bar{t}_v]$.

**Table 3:** Control policy for vaccination of $65+$ first

Age group	Vaccination period	$\%$ of vaccinated within the group	$\%$ of vaccinated
65+	150–213	100$\%$	16.9$\%$
30–65	214–250	39.68$\%$	12.9$\%$

**Table 4:** Control policy for random vaccination

Age group	Vaccination period	$\%$ of vaccinated within the group	$\%$ of vaccinated
18–30	150–250	47.7$\%$	4.2$\%$
30–65	150–250	47.7$\%$	18$\%$
65–80	150–250	47.7$\%$	5.5$\%$
80+	150–250	47.7$\%$	2.1$\%$

**Table 5:** Control policy for optimal vaccination

Age group	Vaccination period	$\%$ of vaccinated within the group	$\%$ of vaccinated
18–30	150–190	100$\%$	10.9$\%$
80+	191–210	100$\%$	4.9$\%$
65–80	211–240	100$\%$	11.2$\%$
30–65	241–250	7.3$\%$	2.8$\%$

The vaccination period is chosen to contain the majority of the infected cases in our experiments and also to demonstrate the differences in the vaccination strategies. Having this period shifted more to the left of the horizon, i.e. vaccination is administered earlier, has an even greater effect of reducing infections and mortality. This is due to the fact that vaccinated people are protected against future infections.

In the experiments the second constraint in (2.17) reads as $\sum _{j=1}^5\bar{v}_j(t)\le 0.003$, i.e. the daily vaccination capability is $0.3\%$ of the population. The value is chosen to represent somewhat realistic constraint on the daily capability of administering vaccines either from supply, personnel considerations or people’s willingness to be vaccinated.

We compare the results from experiments with different case studies. On Tables 3, 4 and 5 are shown the vaccination policies for the different vaccination approaches. During each vaccination period the maximum daily vaccination capacity is administered to the corresponding age group. In the case of random vaccination, Table 4, the vaccination is administered simultaneously to all the groups except the youngest age group, that is why the groups vaccination period coincides with the $[t_v, \bar{t}_v]$ period.

In Table 3 we see that after 63 days of vaccination all elderly people are vaccinated. After that our optimal solution suggests to start vaccinating the age group of $30-65$ years.

Different is the case of the optimal vaccination policy shown in Table 5. The obtained optimal solution suggests to begin the vaccination with the highest contact rate group of individuals $18-30$ years. After the group of young working is fully vaccinated on day 190 the solution switches policy to the people with highest mortality rate ($80+$) and again after this group is vaccinated the vaccination continues with the group of $65-80$ years. The vaccination ends with the vaccination of the rest of the working population, $30-65$ years. We explain that the prioritization of the young working group $18-30$ at the beginning of the vaccination interval is due to the high impact of the contact rate parameter in our model, i.e. although the elderly population has the highest mortality rates, we try to minimize the total number of fatality cases among all groups.

**Table 6:** Control policy for optimal vaccination with reduced 18-30 contact rate

Age group	Vaccination period	$\%$ of vaccinated within the group	$\%$ of vaccinated
30–65	150–210	52.3$\%$	16.6$\%$
80+	211–230	100$\%$	5.2$\%$
65–80	231–250	71.2$\%$	8.1$\%$

**Table 7:** Control policy for optimal vaccination with increased 80+ contact rate

Age group	Vaccination period	$\%$ of vaccinated within the group	$\%$ of vaccinated
80+	150–168	100$\%$	4.7$\%$
65–80	169–208	100$\%$	11.3$\%$
30–65	209–250	43.7$\%$	13.9$\%$

In addition, in Tables 6 and 7, we show optimal solutions obtained when changes are made to the contact rates of group $18-30$ years and group $80+$ years. For the experiment in Table 6 the contact rate factor for group $18-30$ is reduced to the value of group $30-65$ years, i.e. from 1.6209 to 1.2250. Here we can observe that the order of vaccination is changed, the vaccination begins with the group $30-65$ years. In contrast to the previous observed optimal behavior, the vaccination ends before all individuals in the group are vaccinated. This is due to the size of this group which is significantly larger than the rest of the groups. Afterwards the strategy again continues with the vaccination of the two elderly groups.

For the experiment in Table 7 the contact rate factor for group $80+$ is increased again to the value of group $30-65$ years, i.e. from 0.7011 to 1.2250. For this example we observe again changes in the order of vaccination. This time the vaccination begins with group $80+$, and — after all group members are vaccinated — continues with group $65-80$. Finally it ends with the vaccination of group $30-65$.

It should be noted that varying one parameter changes the overall disease evolution over time. The obtained optimal vaccination solutions with respect to the various contact rate factors are compared only with the changed optimal vaccination order.

#### Remark 4.1

Shrinking the vaccination period $[t_v, \bar{t}_v]$ plays also a role for the optimal vaccination. Having a small interval for vaccination prioritizes the most vulnerable, i.e. the elderly population, to be vaccinated first. In our case increasing the end $\bar{t}_v$ of the vaccination horizon preserves the obtained strategy to vaccinate first the group with the highest contact rate.

On Fig. 1 are represented the results for the different vaccination strategies showing the total percentage of infected individuals and the total percentage of mortality cases for all groups. From the left plot of Fig. 1 one can observe that the random vaccination strategy is more efficient in reducing the overall infected percentage compared to strategy of vaccination the elderly population of $65+$ people. Although, the case fatality rates of age groups $65-80$ and $80+$ is significantly higher than the other groups, we can observe that by prioritizing the vaccination of the $65+$ the total mortality is comparable to the random vaccination. In the case of the optimal vaccination policy (Table 5) on Fig. 1 it’s shown that this strategy is superior to the other cases, both in reducing the number of infected individuals and reducing the overall fatality cases.

**Fig. 2:**
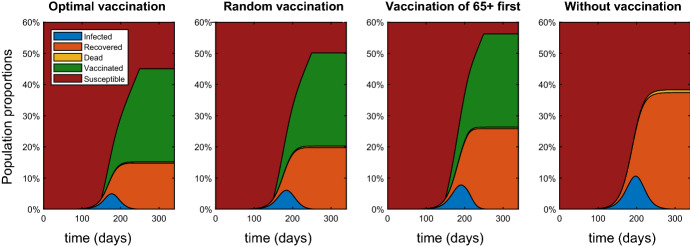
Comparison between the different vaccination strategies with respect to the population compartments

During the vaccination period the same number of vaccines are administered to the population with different results. Figure 2 shows the evolution of the population compartments during the evolution of the disease. One can observe the changes of the levels not only for the infected individuals but also for the recovered. After the vaccination period [150, 250], between $43\%$ and $58\%$ of the population is vaccinated or recovered from the disease depending on the strategy. The rest of the population consist mainly of susceptible individuals, but small portion of infected individuals (that may show no symptoms) remains. This means one can continue to vaccinate even after the considered vaccination period.

## Discussion

In this study, we build on a previously developed epidemiological model by including vaccination strategies. Several vaccination options are investigated, such as random vaccination or prioritization of population groups according to their age classification. The latter is of particular interest for the application of the model to COVID-19. It is well known that age is a critical population vulnerability factor. The risk of severe illness with COVID-19 increases with age where the elderly population is at highest risk (Wu et al. 2020). In an optimal control framework we develop vaccination policies, that is, the allocation of the available vaccination capacities to the susceptibles to be vaccinated. Control objectives were chosen here based on the most desirable effects such as to keep the number of hospitalizations such as it does not exceed the capacity of the hospital and therefore does not overwhelm the health care system. Another control goal may be to minimize the number of fatalities. This was also the control objective in the COVID-19 case study.

We formulate the model for random vaccination with heterogeneous populations. Other options such as mixing with different contact rates between and within population groups such as elderly, supporting nurses, physicians and the general population could be incorporated in the model in a straight forward way. While any relaxation of the assumptions and expansion of the model is mathematically doable, the availability of corresponding data is rather difficult. The epidemiological optimal control policy is derived from an optimal control problem for a specific class of of integro-differential equations system with certain characteristics such as control-affinity. Its analysis indicates practical decisions such as vaccination of one group at a time.

The numerical results are based on plausible vaccination strategies for the case of COVID-19. The random vaccination is applied to the population with the exception of age group 0-18 since the latter group is by far the least vulnerable to develop severe disease although it contributes to transmission. In another scenario we prioritize vaccination of the elderly population, a strategy that has been widely followed. Finally, we calculated the optimal vaccination strategy. In comparison to an non-vaccination scenario where the epidemic runs without intervention, vaccination in whatever scenario had a clear positive effect in reducing the number of cases and shortening the duration of the outbreak. In the scenario of prioritization of the elderly the model results show that the optimal solution continues with the age group $30-65$. Interestingly, in the optimal vaccination policy vaccination begins with the population groups having the highest contact rates. This may have implications when designing the vaccination strategy. If other restrictions such as ethical reasons do not speak against changing the order to an optimal scenario. Thus, a clinically advisable scenario in not necessarily also epidemiologically optimal. Clearly, changes of the contact rates may lead to changes in the optimal order at which different age groups should be vaccinated.

Although the presented model can be easily extended in several directions (e.g. by including additional compartments, medication, imperfect effect of vaccination, etc.), there are substantial limitations to its applicability. Of course, as for all complex epidemiological models, a main problem is the data availability. In addition, we assume permanent immunity, which may be plausible only on the short run. Consideration of waning immunity (after vaccination or disease) is a subject of a paper in preparation by the same authors. In addition, we mention that the control strategies considered in this paper, including the optimal ones, may not be fully practical. Such difficulties arise in particular with frequently changing policies or policies requiring vaccination of all members of a group. Nevertheless, the information provided by such strategies gives a hint for what policy should be targeted by the health care authorities.

## Data Availability

All data used in the manuscript are referenced and publicly available.

## Footnotes

1 apart of changes in the contact rates due to restriction policies (social distancing), which are not involved in the present paper

2 Here we consider a time-invariant upper bound just for convenience.

## Declarations

### Disclaimer

The views expressed are purely those of the writer (NIS) and may not in any circumstance be regarded as stating an official position of the European Commission.

## References

[CR1] Acemoglu D, Chernozhukov V, Werning I, Whinston MD (2000) Optimal targeted lockdowns in a multi-group SIR model. National Bureau of Economic Research, Working Paper 27102, May, 2000. URL http://www.nber.org/papers/w27102

[CR2] Bhattacharaya K, Ghosh A, Monsivais D, Dunbar RIM, Kaski K (2016). Sex differences in social focus across the life cycle in humans. Royal Society Open Science.

[CR3] Bloom DE, Kuhn M, Prettner K (2000) Modern infectious diseases: macroeconomic impacts and policy responses. PGDA Working Paper No. 187, August, 2000. URL http://www.hsph.harvard.edu/pgda/working/

[CR4] Bubar K, Reinhold K, Kissler SM, Lipsitch M, Cobey S, Grad YH, Larremore DB (2021). Model-informed COVID-19 vaccine prioritization strategies by age and serostatus. Science.

[CR5] Caulkins JP, Grass D, Feichtinger G, Hartl RF, Kort PM, Prskawetz A, Seidl A, Wrzaczek S (2021). The optimal lockdown intensity for COVID-19. J Math Econ.

[CR6] Davis NG, Klepac P, Liu Y, Prem K, Jit M, CMMID COVID-19 working group, Eggo RM, (2020) Age-dependent effects in the transmission and control of COVID-19 epidemics Nat. Med. 26:1205–1211 10.1038/s41591-020-0962-9 32546824

[CR7] Jentsch PC, Anand M, Bauch CT (2021). Prioritising COVID-19 vaccination in changing social and epidemiological landscapes: a mathematical modelling study. Lancet Infect Dis.

[CR8] Johansson MA, Quandelacy TM, Kada S, Vemkanda Prasad P, Steel M, Brooks JT, Slayton RB, Biggerstaff M, Butler JC (2021). SARS-CoV-2 transmission from people without COVID-19 symptoms. JAMA Netw Open.

[CR9] Kovacevic RM, Stilianakis NI, Veliov VM (2022). A distributed optimal control model applied to COVID-19 pandemic. SIAM J Control Opt.

[CR10] Matrajt L, Eaton J, Leung T, Brown ER (2021). Vaccine optimization for COVID-19: Who to vaccinate first?. Sci Adv.

[CR11] Moore S, Hill EM, Tildeslay MJ, Dyson L, Keeling MJ (2021). Vaccination and non-pharmaceutical interventions for COVID-19: a mathematical modelling study. Lancet Infect Dis.

[CR12] Onder G, Rezza G, Brusaferro S (2020). Case-fatality rate and characteristics of patients dying in relation to COVID-19 in Italy. JAMA.

[CR13] Signorelli C, Odone A (2020). Age-specific COVID-19 case-fatality rate: no evidence of changes over time. Int J Public Health.

[CR14] Statistics Austria. Bevölkerung zu Jahresbeginn 2002-2020 nach fünfjährigen Altersgruppen und Geschlecht. https://www.statistik.at/web_de/statistiken/menschen_und_gesellschaft/~bevoelkerung/bevoelkerungsstruktur/bevoelkerung_nach_alter_geschlecht/index.html

[CR15] Verity R, Okell LC, Dorigatti I, Winskill P, Whittaker C, Imai N (2020). Estimates of the severity of coronavirus disease 2019: A model-based analysis. Lancet Infect Dis.

[CR16] Wu C, Chen X, Cai Y, Xia J, Zhou X, Xu S (2020). Risk factors associated with acute respiratory distress syndrome and death in patients with coronavirus disease 2019 pneumonia in Wuhan. China JAMA Intern Med.

